# First Complete Genome Sequencing of a Pigeonpox Virus Strain from Mainland China and Preliminary Evaluation of Its Attenuated Potential

**DOI:** 10.3390/vetsci13040393

**Published:** 2026-04-17

**Authors:** Yifan Zhu, Baolichen Zhang, Zhongshu Ji, Jingliang Su, Jianyu Chang, Kai Fan

**Affiliations:** 1 College of Veterinary Medicine, China Agricultural University, Beijing 100193, China; b20213050451@cau.edu.cn (Y.Z.); zblc@cnilas.org (B.Z.); 2025jzs@sina.com (Z.J.); suzhang@cau.edu.cn (J.S.); 2 Institute of Laboratory Animals Science, Peking Union Medical College (Chinese Academy of Medical Sciences), Beijing 100730, China

**Keywords:** pigeonpox, prevalence, genome, attenuation, vaccine efficacy

## Abstract

Pigeonpox is a significant infectious disease caused by *Pigeonpox virus* (PPV), which severely impacts the pigeon industry. Current control methods primarily rely on heterologous vaccines, such as those derived from avian poxviruses, but their protection is limited, creating an urgent need for the development of a specific vaccine. In this study, 720 samples collected from several regions of China between 2022 and 2023 were tested for PPV, followed by virus isolation, identification, and genetic evolutionary analysis. Based on these findings, complete genome sequencing and attenuation of the representative BJ-02 isolate were conducted, and the potential of this strain as a candidate for an attenuated vaccine was preliminarily evaluated. The survey showed PCR positive rates of 9.05%, 16.11%, and 12.50% in samples from Beijing, Guangdong, and Hainan, respectively. Six viral strains were isolated, all of which produced typical lesions on chorioallantoic membranes (CAM) and chicken embryo fibroblasts (CEF). Phylogenetic analysis based on the P4b gene revealed that the six viruses clustered within the same evolutionary branch, closely related to PPV and penguinpox virus strains from South Africa, India, and Taiwan, China. Complete genome sequencing of the BJ-02 strain showed its genomic structure to be similar to that of other fowlpox viruses, with some differences. After serial passage in CAM, PEF and CEF, the BJ-02 SD55 high-passage strain adapted well to in vitro culture, exhibited significantly reduced pathogenicity in chicken embryos and pigeons, and showed no reversion to virulence after five consecutive back-passages. Animal immunization tests demonstrated that the BJ-02 SD55 suspected attenuated strain induced specific antibodies and provided 100% protection against challenge with the virulent strain. In conclusion, PPV is widely prevalent in China. The BJ-02 strain, successfully isolated and attenuated through serial passage, demonstrates excellent immunogenicity and high safety, making it a promising candidate for a specific pigeonpox vaccine. Additionally, the complete genome characterization of BJ-02 contributes to the avian poxvirus genome database and provides critical data to support research on viral pathogenesis and the development of viral vector vaccines for avian and potentially mammalian species.

## Simple Summary

Pigeonpox is a significant infectious disease caused by the *Pigeonpox virus* (PPV), which severely impacts the pigeon industry. Current control methods primarily rely on heterologous vaccines, such as those derived from avian poxviruses, but their protection is limited, creating an urgent need for the development of a specific vaccine. This study first investigated the epidemiological status of pigeon pox in parts of China, revealing its distribution across multiple provinces and successfully isolating several viral strains from infected pigeons. We performed whole-genome sequencing on a representative strain to obtain detailed genomic data. Through laboratory cultivation techniques, we attenuated the virulence of the strain, ensuring it no longer causes disease while maintaining its immunogenic properties. Experimental results demonstrate that this attenuated strain is safe and induces robust immunity in pigeons, providing 100% protection. This research not only offers a highly promising candidate for a specialized pigeon vaccine—helping to mitigate economic losses in the industry—but also provides a vital scientific foundation for the future development of viral vector vaccines for other avian species and potentially even humans by deciphering the virus’s genetic composition.

## 1. Introduction

Fowlpox is a common viral disease caused by Avipoxvirus (APV), which is widely prevalent in both domestic and wild bird populations worldwide [1,2]. Infected birds typically develop proliferative and necrotic lesions on featherless areas of the body. When the infection is complicated by the presence of avian reticuloendotheliosis virus gene sequences or secondary bacterial or fungal infections, it can result in decreased egg production, impaired growth, and, in severe cases, death [3,4,5]. Avipoxvirus is a double-stranded DNA virus with a genome size ranging from 260 kb to 365 kb, making it the largest and most structurally complex member of the Chordopoxvirinae subfamily within the Poxviridae family [6]. Currently, phylogenetic studies of Avipoxvirus primarily rely on whole genome sequencing and analysis of conserved genes. However, due to limitations in sequencing technologies and the scope of research, the host range, genetic diversity, and evolutionary mechanisms of this virus remain inadequately understood [7]. Phylogenetic analysis based on the conserved P4b gene classifies APV into three major clades: clade A (avianpox-like), clade B (canarypox-like), and clade C (parrotpox-like). Clade A is further divided into seven subclades (A1–A7), while clade B consists of three subclades (B1–B3) [8,9,10]. APV strains originating from different hosts exhibit significant differences in antigenicity and gene sequences, suggesting that these strains may have undergone complex genetic recombination over time to adapt to diverse host environments [10,11].

Since APV replicates and transcribes in the host cytoplasm, and its genome does not enter the nucleus, the risk of integration into the host genome is avoided. Furthermore, APV is capable of accommodating and efficiently expressing multiple exogenous genes but cannot complete its replication cycle in non-avian hosts. As a result, avipoxviruses have been evaluated as ideal vectors for genetic engineering vaccines in both avian and mammalian species [12,13,14,15]. Recombinant vaccines based on avipox vectors have been widely used in veterinary medicine, and some vaccines employing avipoxviruses as vectors have also been explored for human disease prevention [16,17,18,19].

Pigeonpox is a contagious disease caused by the *Pigeonpox virus* (PPV), a member of the Avipoxvirus genus, capable of infecting pigeons of various breeds and ages. Infected pigeons often show symptoms such as reduced feed intake, growth retardation, emaciation, and, in severe cases, death. In squab farming, the formation of pock scabs significantly affects the quality of the skin, leading to direct economic losses [20]. In recent years, infections with PPV in China have been on the rise. Due to climate change and increasing temperatures, the active period for vectors such as mosquitoes has extended, and in some regions, the disease has become endemic throughout the year. This has had a significant impact on both the racing and meat pigeon industries. Current clinical prevention and control primarily rely on heterologous live vaccines derived from Fowlpox virus. However, due to antigenic differences between Fowlpox and PPV, these vaccines offer limited protection, and outbreaks still occur even among vaccinated flocks [21].

Research into the genomics of avian poxviruses is still in its early stages. As of early 2025, only three complete PPV genome sequences have been published in the international GenBank database: ON375849.1 and NC024447.1, previously uploaded, and PV250209, which was recently submitted by Taiwan. However, no complete PPV genome sequences have been recorded from mainland China, limiting a deeper understanding of the virus’s genomic characteristics, genetic evolution, and compatibility of vaccine strains circulating within the country. The lack of data is primarily due to the technical difficulties and high costs associated with whole-genome sequencing of PPV, which is one of the avian poxviruses with the largest genome. This limitation has resulted in an extremely narrow pool of available genomic data. This research gap not only hinders the understanding of the molecular pathogenesis of PPV but also impedes the development of highly effective, specific vaccines. Therefore, conducting whole-genome sequencing of newly isolated strains, particularly those representing indigenous epidemic strains in China, is of significant scientific and practical value.

This study presents an epidemiological survey, viral isolation, and identification of PPV from clinical samples collected between 2022 and 2023 in Beijing, Guangdong, and Hainan provinces of China. Based on these findings, representative isolates were selected for whole-genome sequencing to analyze their genomic structure. In addition, in vitro serial passages were performed to evaluate their attenuation potential and immunogenicity as candidate strains for live attenuated PPV vaccines. The study aims to shed light on the current epidemiological status of PPV in certain regions of China, fill the gap in whole-genome data for PPV from mainland China, and provide a critical reference for the development of specific PPV vaccines and the optimization of avian poxvirus vector systems.

## 2. Materials and Methods

### 2.1. Sample Collection

Samples for testing were collected from breeding and homing pigeons at pigeon farms and local live poultry markets in Beijing, Guangdong, and Hainan. Farmers at the sampling locations reported that some pigeons exhibited signs of PPV infection. The sampled population had not been vaccinated against avian poxvirus and included squabs (under 4 months of age), juvenile pigeons (4 months to 1 year), and adult pigeons (over 1 year), with an average age under 12 months, representing a population susceptible to PPV. Sampling was primarily conducted between July and September, which coincided with the peak activity period of mosquitoes and insects during the summer, as well as the high-incidence period of PPV outbreaks. A total of 720 pigeons were sampled, with one oropharyngeal swab collected per bird, totaling 720 swabs. If visible skin lesions or lesions around the eyes or beak suggestive of PPV infection (e.g., pox scabs) were observed, suspected scabs or skin samples from the lesion sites were collected. In total, 108 lesion samples were collected from 108 individual pigeons.

### 2.2. Sample Processing

The head of each throat swab was submerged into a sterile Eppendorf tube containing 500 µL of sterile PBS. The mixture was vortexed for 15 s and then centrifuged at 12,000 rpm for 1 min to collect the supernatant. For pock crusts and skin samples from lesion sites, the samples were ground using a grinder, then sterile PBS was added, and high-speed centrifugation was performed to collect the supernatant. All samples were stored at −80 °C for later use. Viral nucleic acid extraction was carried out following the EasyPure^®^ Viral DNA/RNA Kit package instructions. Extracted DNA was stored at −20 °C for future use.

### 2.3. PCR-Based Identification

Primers were designed using Primer5.0 software based on the published conserved sequence of the P4b gene of PPV strains. The upstream and downstream primers were P4b-F: 5′-TGGTTTACTACTACGAAGACGC-3′ and P4b-R: 5′-CCTTGAAAAGAGTCATAATG-3′, respectively. PCR amplification was performed using the extracted DNA as a template, following standard procedures.

### 2.4. Virus Isolation and Culture

The virus samples that tested positive from three regions were labeled as BJ-01, BJ-02, GD-01, GD-02, HN-01, and HN-02. These six samples were ground, centrifuged, and the supernatant was collected. Double antibiotics were added to a final concentration of 5000 IU/mL, and the mixture was treated overnight at 4 °C. Afterward, the virus solution was inoculated onto the chorioallantoic membranes of healthy 9–11-day-old chicken embryos, with 0.2 mL per embryo, and the air sac facing upward. The embryos were incubated in a 37 °C incubator for 5 days. Embryos that died within 24 h were discarded daily. After 5 days, the embryos were stored at −4 °C for 4–6 h, and then the eggshells were opened using sterile tools to expose the chorioallantoic membrane, with or without lesions. The membrane was cut out and placed into sterile Eppendorf tubes, then subjected to three freeze–thaw cycles at −80 °C to release the virus. The same method was used for three consecutive passages in 9–11-day-old chicken embryos, and the chorioallantoic membranes with lesions were preserved at −80 °C.

In parallel, chicken embryo fibroblasts were used to culture the isolated virus. The chorioallantoic membranes from infected chicken embryos were subjected to three freeze–thaw cycles at −80 °C, followed by low-temperature high-speed centrifugation to remove impurities. Sterile PBS was added, and the mixture was filtered through a 0.22 µm bacterial filter. The processed viral stock solution was inoculated onto confluent monolayers of chicken embryo fibroblasts. After 2 h of adsorption, the viral inoculum was removed, and the cells were washed twice with sterile PBS. Maintenance medium was added, and the plates were incubated at 37 °C with 5% CO_2_ for 4–6 days.

### 2.5. Phylogenetic Analysis of P4b

Viral genome DNA was extracted from chorioallantoic membranes of chicken embryos obtained through passage. The P4b gene was amplified using the appropriate primers and 2% agarose gel electrophoresis. Target fragments were recovered from the gel. According to the pEASY-T1 Simple Cloning Kit instructions, genes of interest were ligated into the pEASY-T1 vector, and DH5α competent cells were transformed using standard procedures. Transformed cells were plated onto Amp-resistant LB plates, incubated overnight, and colonies were subjected to PCR identification. Positive colonies were cultured, and 500 µL of culture was sent to a sequencing company for P4b gene sequencing. The obtained P4b gene sequence was aligned with 22 publicly available P4b sequences from different avian poxviruses in the GenBank database. Phylogenetic analysis was performed using MEGA11 software (version 10.2.6).

### 2.6. Complete Sequence and Analysis

Chorioallantoic membrane tissue samples, which tested positive for PGPV by PCR, were sent to Sangon Biotech (Shanghai) Co., Ltd., (Shanghai, China) for high-throughput sequencing. The raw data were compared to the NCBI database with BBMap (v38.51) to remove ribosomal RNA, host genome, and bacterial contaminant sequences. SOAPdenovo (version 2.04) and SPAdes software (v3.14.1) were used for *de novo* genome assembly based on the De Bruijn graph algorithm and multi-gradient k-mer parameters, and complete scaffolds were generated through cycle filling. The assembly results were aligned against the virus-NT database by BLAST (NCBI) to determine the species origin. Prokka (v1.14.5) software was used for genome annotation, combined with Prodigal (v0.9) to predict coding genes, and HMMER3.1 was utilized to identify gene families based on conserved domains. DNAMAN software (version 9) was used for sequence similarity alignment, and the biological characteristics of the BJ-02 strain were analyzed combined with protein function prediction.

### 2.7. Attenuation of the BJ-02 Strain

The virus was serially passaged for attenuation in 9–11-day-old SPF chorioallantoic membranes, pigeon embryo fibroblasts, and chicken embryo fibroblasts. The specific passage protocol was as follows: the BJ-02 isolate was first passaged continuously for 5 generations in SPF chorioallantoic membranes, with each passage inoculated with 0.2 mL of tissue supernatant harvested from the previous passage, which was subjected to repeated freeze–thaw and filtration treatment, for a total of 5 chicken embryos. Then it was passaged 10 times consecutively on pigeon embryo fibroblasts, and finally passaged more than 40 times on chicken embryo fibroblasts. Each cell passage was inoculated with the supernatant of the culture medium harvested from the previous passage and subjected to repeated freeze–thaw and centrifugation treatment. In total, 1 mL of virus solution was used to infect each T25 culture flask to ensure sufficient virus particles to infect the cells. Three T25 culture flasks were passaged simultaneously, and a negative control was included.

Viral stocks from passages 3, 8, 18, 38, 50, and 55 were inoculated into SPF chorioallantoic membranes to observe and compare the severity of lesions on the chorioallantoic membranes induced by viruses at different passage levels after incubation for 5 days. Chicken embryo fibroblasts were infected with the low-passage strain BJ-02 SD03 and the high-passage strain BJ-02 SD55, respectively, covered with 1 mL of nutrient agarose (1% LMA + 1 × MEM + 2% FBS + 1% P/S), and cultured upside down at 37 °C and 5% CO_2_ for 6 days. Plaque size differences were observed and measured after staining with 0.01% neutral red. Meanwhile, chicken embryo fibroblasts were infected with the two passages of virus, and infected cell samples were collected every 24 h. Virus titers at each time point were measured, growth curves were plotted, and the differences in viral titers and in vitro proliferation trends between the two strains were analyzed.

### 2.8. Safety Assessment of BJ-02 SD55

Low-generation strain BJ-02 SD03 and high-generation strain BJ-02 SD55 were inoculated into 10 healthy 30-day-old pigeons using the wing-web method. The occurrence of specific pox lesions at the inoculation site and other featherless areas of the body was observed daily for 28 days to assess the difference in pathogenicity between the low-generation and high-generation strains. Meanwhile, 5 healthy 30-day-old non-immunized pigeons were inoculated with the high-generation strain BJ-02 SD55 by the same method, with each pigeon receiving 0.2 mL of tissue suspension. When obvious lesions appeared at the inoculation site, skin or pox lesions were collected, processed, and re-inoculated into 5 pigeons. This process was repeated 5 times. The disease incidence in the pigeons was observed and recorded to assess the stability of the virulence of the high-generation strain BJ-02 SD55 in the pigeons. All the pigeons used in the above and subsequent experiments were purchased from a fixed unit, and the experiments were only conducted after the pigeons were assessed as having negative serum antibodies using a plaque reduction and neutralization test before the experiments began.

### 2.9. Antibody Dynamics and Challenge Protection of Strain BJ-02 SD55

Twenty healthy, 30-day-old non-immune squabs were selected and randomly divided into two groups (10 squabs per group). The immunized group was inoculated via the wing-web method with the attenuated strain BJ-02 SD55 (10^5^ PFU/bird), while the control group was injected with sterile normal saline (0.2 mL/bird). Blood samples were collected from the wing vein on days 7, 14, 21, and 28 post-immunization. The serum was separated, and antibody dynamics were monitored via Western blot, ELISA, and plaque reduction neutralization tests. On day 28 post-immunization (58-day-old), the squabs were challenged via the wing-web method on the other side of the wing with the virulent strain BJ-02 SD03 (10^5^ PFU/bird). Antibody levels were detected every 7 days post-challenge, and pock lesions at the inoculation site and other featherless body surfaces were observed and recorded daily. The immunogenicity and protective efficacy of the high-passage strain BJ-02 SD55 in combination with antibody levels was evaluated. The detailed experimental protocol is shown in Table 1.

### 2.10. Reagents, Consumables, and Equipment

The EasyPure^®^ Viral DNA/RNA Kit (ER201-01) and pEASY^®^-T1 Simple Cloning Kit (CT111-02) were purchased from TransGen Biotech Co., Ltd. (Beijing, China). SPF chicken embryos were obtained from Beijing Boehringer Ingelheim Vital Biotechnology Co., Ltd. (Beijing, China). DH5α competent cells (TSC-C14) were purchased from Tsingke Biotechnology Co., Ltd. (Beijing, China). Cell culture flasks were purchased from Wuxi NEST Biotechnology Co., Ltd. (Wuxi, China). Low-melting-point agarose (A8350), MEM medium (41500), 0.33% neutral red staining solution (G1310), and penicillin-streptomycin solution (P1400) were obtained from Beijing Solarbio Science & Technology Co., Ltd. (Beijing, China). Fetal bovine serum (BMC1020) was purchased from Wuhan Yakeyin Biotechnology Co., Ltd. (Wuhan, China). A PCR thermal cycler (T100; Bio-Rad Laboratories, Inc., Hercules, CA, USA) and a tissue homogenizer (Ningbo Xinzhi Biotechnology Co., Ltd., Ningbo, China) were used in this study. All other reagents and consumables were routine laboratory-grade products.

## 3. Results

### 3.1. Infection with Pigeonpox Virus

**Figure 1:**
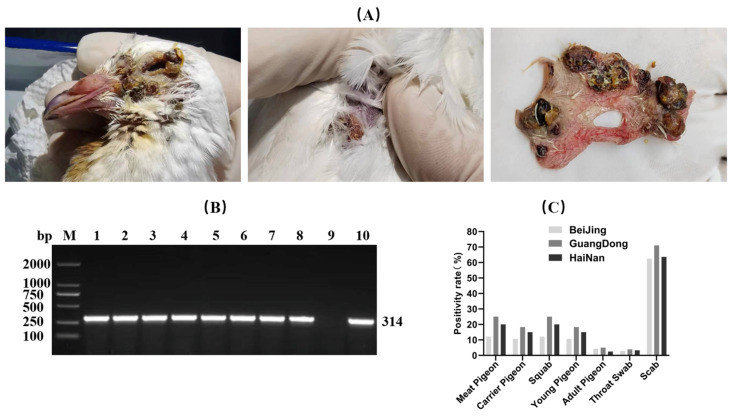
Investigation of *pigeonpox virus* infection. (**A**) Pox lesions observed on the body surface of infected pigeons (**B**) PCR testing results of selected samples, M: DNA Marker 1–8: Samples 9: Negative control, 10: Positive control (**C**) Summary chart categorizing and presenting the positive rates of all samples.

Each pigeon was tested using either a throat swab or both a throat swab and lesioned skin samples (e.g., scabs). If either result was positive, the pigeon was considered infected with PPV. The results indicated that PPV infection was present to varying degrees in pigeon flocks across the three regions, with overall positive rates of 9.05%, 16.11%, and 12.50%, respectively. Regarding sample types, the highest viral detection rate was observed in scab samples, exceeding 60% in all three regions. There was no significant difference in positive rates between different pigeon breeds (meat pigeons versus homing pigeons), but infection rates were higher in young and juvenile pigeons compared to adult pigeons (Figure 1).

### 3.2. Virus Isolation and Cell Culture

**Figure 2:**
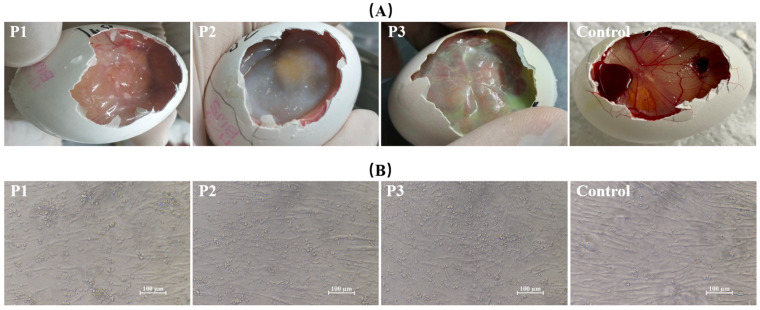
Isolation and culture of *pigeonpox virus*. (**A**) Lesions observed in chorioallantoic membranes from different passages of the isolate. (**B**) Cytopathic effects in chicken embryo fibroblast (CEF) cells following infection with the isolate.

After inoculation of SPF chicken embryos, the chorioallantoic membrane of the first-generation embryos showed mild thickening with scattered, opaque, off-white pock lesions. By the second generation, the allantoic membrane exhibited significant edema and thickening, with large opaque gray-white areas. By the third generation, the allantoic membrane became markedly thickened and appeared light red, easily lifted with tweezers (Figure 2A).

At 72 h post-inoculation of chicken embryo fibroblast (CEF) cells with the pigeonpox isolate, marked changes in cell morphology were observed, including cell shrinkage, rounding, increased refractivity, and widened intercellular spaces, typical cytopathic effects. As the culture time progressed, the cytopathic effects intensified, with an increasing number of cells dying, detaching, and floating in the medium (Figure 2B).

### 3.3. Phylogenetic Analysis of P4b

**Figure 3:**
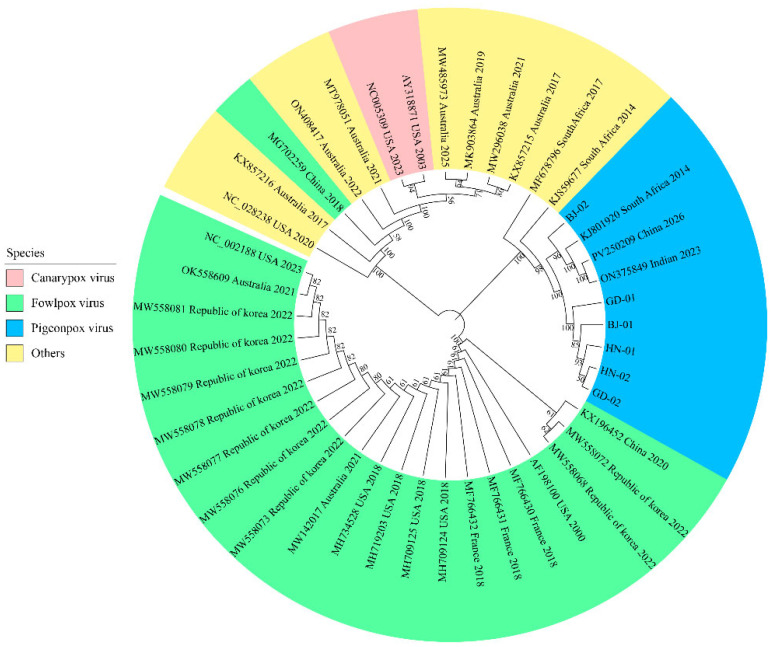
Phylogenetic tree based on the P4b gene of pigeonpox isolates.

Six positive isolates were selected and designated BJ-01, BJ-02, GD-01, GD-02, HN-01, and HN-02. The conserved sequences of the P4b gene from these six isolates were sequenced and compared with P4b gene sequences of 37 fowlpox viruses from different species retrieved from the NCBI database for phylogenetic analysis. The phylogenetic tree is divided into two major clades, with the *pigeonpox virus* isolates clustering in the first major clade. These isolates were closely related to three *pigeonpox viruses* from India, South Africa, and Taiwan, China, and one penguinpox virus from South Africa. However, they were distantly related to fowlpox viruses isolated from chickens, magpies, and canaries (Figure 3).

### 3.4. Complete Sequence and Analysis

**Figure 4:**
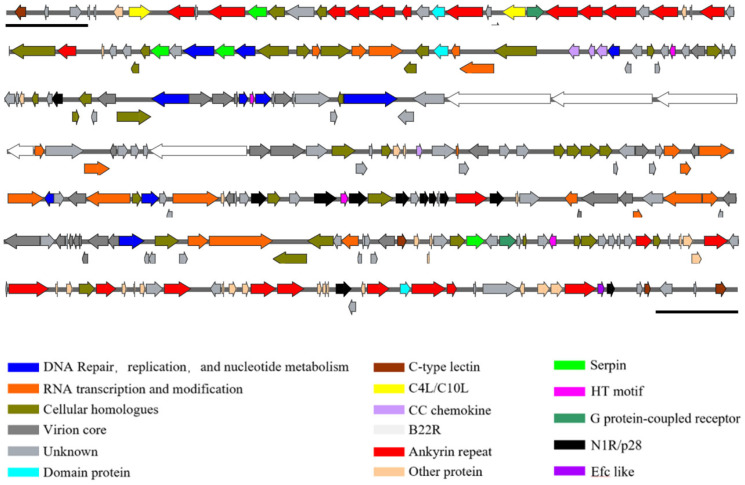
Complete genome map of isolate BJ-02.

The complete genome of the BJ-02 isolate was identified as a contiguous sequence of 280,269 bp and has been submitted to the GenBank database under the accession number PX219506. Its total length was comparable to that of two previously published *pigeonpox virus* full-length sequences, though shorter than other fowlpox virus genomes. Due to limitations of the sequencing method, the assembled complete sequence did not include the terminal hairpin loop structure. Therefore, the first nucleotide was designated as the leftmost nucleotide of the full-length sequence for subsequent annotation and analysis. The BJ-02 genome consists of two terminal inverted repeat (ITR) sequences, each 4691 bp in length, flanking a central coding region. This genomic structure was consistent with that of other fowlpox viruses from different species. The A + T content was 70%, with some regions of the genome slightly deviating from this value. Using Prokka software, a total of 270 open reading frames (ORFs) were identified (listed in Supplementary Table S1), and a complete genome map was constructed (Figure 4). These genes collectively cover 90% of the coding density, encoding structural or functional proteins ranging from 38 to 1938 amino acids.

The BJ-02 genome sequence was aligned with other published fowlpox virus genome sequences from different species available in NCBI using DNAMAN software. The results showed that BJ-02 had high sequence homology with *pigeonpox virus* genomes isolated from South Africa (NC_024447.1) and India (NC_036582.1) (>95%), while it showed the lowest similarity to turkeypox virus (70.29%) and intermediate similarity to other avian poxvirus genomes (72.56%–89.27%). This suggests a certain degree of conservation among avian poxvirus genomes, with some differences. The genome map compares BJ-02 with its closest orthologs. Among the 270 ORFs, 226 were annotated as complete genes, 15 as truncated genes, and 29 as fragmented forms of larger homologous genes. Similar to other sequenced fowlpox virus genomes, the BJ-02 genome contains 179 conserved genes, including core conserved genes and genes located in the variable terminal regions. The number of genes in the BJ-02 gene family is slightly different from that in FeP2 (NC_024447.1). There are 23 ankyrin repeat family proteins, fewer than in FeP2 (26 proteins), with missing full-length ankyrin repeat genes mostly present in truncated forms. Three V-type Ig domain family proteins were present in BJ-02, fewer than in FeP2 (four proteins), with the missing gene present in a fragmented form. The number of proteins in the remaining gene families is consistent with that in the FeP2 genome. Detailed alignment data can be found in Supplementary Table S2. Furthermore, no sequences similar to avian reticuloendotheliosis virus (REV), present in fowlpox or other avian poxvirus genomes, were detected in the whole genome of the BJ-02 sequence.

### 3.5. Attenuation and Verification of Strain BJ-02

**Figure 5:**
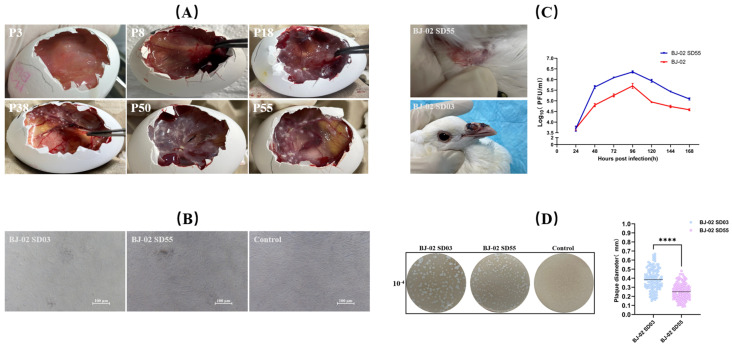
Test result on attenuation of the BJ-02 Isolate. (**A**) Lesions in chorioallantoic membranes caused by a virus from different passage numbers during serial passage; (**B**) cellular pathological changes caused by infections with the viruses from BJ-02 SD03 and BJ-02 SD55; (**C**) lesions in pigeons inoculated with BJ-02 SD03 and BJ-02 SD55 and growth curves on CEF. (**D**) Comparison of plaque morphology between the BJ-02 SD03 and BJ-02 SD55. Statistical analysis was performed using two-way ANOVA and Sidak multiple comparison test (*p* < 0.0001 for ****).

During continuous passages, virus harvests from different passage numbers were inoculated onto chicken embryo chorioallantoic membranes. As the number of passages increased, the severity of lesions on the chorioallantoic membrane gradually decreased. Infection with low-passage-number virus caused significant edema and thickening of the membrane, with pock lesions fusing into sheets. In contrast, from passage 40 onwards, the virus caused scattered off-white pock lesions, evenly distributed or primarily concentrated at the inoculation site (Figure 5A). To compare the in vitro replication characteristics of low- and high-passage viruses, growth curves were determined using chicken embryo fibroblasts (Figure 5B). Both viruses exhibited similar replication trends, with viral titers peaking at 96 h post-infection, followed by a gradual decline. Notably, the peak titer of the passage 55 virus was slightly higher than that of the low-passage virus, suggesting that the virus adapted to the in vitro culture environment through continuous cell passaging and demonstrated improved replication capacity in chicken embryo fibroblasts.

Plaque morphology was assessed on high-density chicken embryo fibroblasts following nutrient agar overlay culture and neutral red staining, with statistical analysis performed using ImageJ software (http://imagej.org) (Figure 5D). The results showed that the diameter of the plaques formed by BJ-02 SD55 high-passage virus was slightly smaller than that of BJ-02 SD03 low-passage virus, suggesting that the virulence of the virus may have decreased after continuous in vitro culture and passage.

To further evaluate the changes in pigeons pathogenicity of viruses from these two generations, 30-day-old squabs were inoculated with virus solutions from the BJ-02 SD03 low-passage and BJ-02 SD55 high-passage respectively. The results showed that the plaque diameter formed by the passage 55 virus was slightly smaller than that of the low-passage virus, indicating reduced viral virulence after continuous cell passaging.

To evaluate changes in animal pathogenicity between the original virus and the attenuated strain, both virus stocks were inoculated into 30-day-old young pigeons. After inoculation with the high-passage virus, pock lesions appeared only at the inoculation site and completely regressed within 21–28 days. The pigeons remained healthy, with no abnormalities observed at other body sites. In contrast, pigeons inoculated with the low-passage virus developed large pock lesions at the inoculation site within 14 days, accompanied by hemorrhage and crusting. These lesions gradually spread to areas such as the beak, periorbital region, and claws, forming multiple black pox lesions. Severely affected pigeons had lesions blocking the nostrils, which impaired respiration, and showed reduced mental status, decreased appetite, and water intake, with some individual deaths occurring during the observation period (Figure 5C).

Our results demonstrate that over five consecutive passages in pigeons, no typical clinical symptoms were observed beyond localized pox at the inoculation site (which resolved within 28 days). The overall health of the subjects remained stable, confirming that the BJ-02 SD55 strain maintains excellent genotypic and phenotypic stability in the host. Detailed results are shown in Table 2.

**Table 2:** The results of reversion to virulence test for BJ-02 SD55.

Passage	Pigeons with Symptom	Symptom
P1 (BJ-02 SD55)	5/5	Within 7–12 days, the inoculation site developed pox; within 28 days, scabs formed and fell off, and the skin returned to normal; other areas remained normal
P2 (BJ-02 SD56)	5/5	Within 7–12 days, the inoculation site developed pox; within 28 days, scabs formed and fell off, and the skin returned to normal; other areas remained normal
P3 (BJ-02 SD57)	5/5	Within 7–12 days, the inoculation site developed pox; within 28 days, scabs formed and fell off, and the skin returned to normal; other areas remained normal
P4 (BJ-02 SD58)	5/5	Within 7–12 days, the inoculation site developed pox; within 28 days, scabs formed and fell off, and the skin returned to normal; other areas remained normal
P5 (BJ-02 SD59)	5/5	Within 7–12 days, the inoculation site developed pox; within 28 days, scabs formed and fell off, and the skin returned to normal; other areas remained normal

### 3.6. Antibody Dynamics and Challenge Protection of Strain BJ-02 SD55

**Figure 6:**
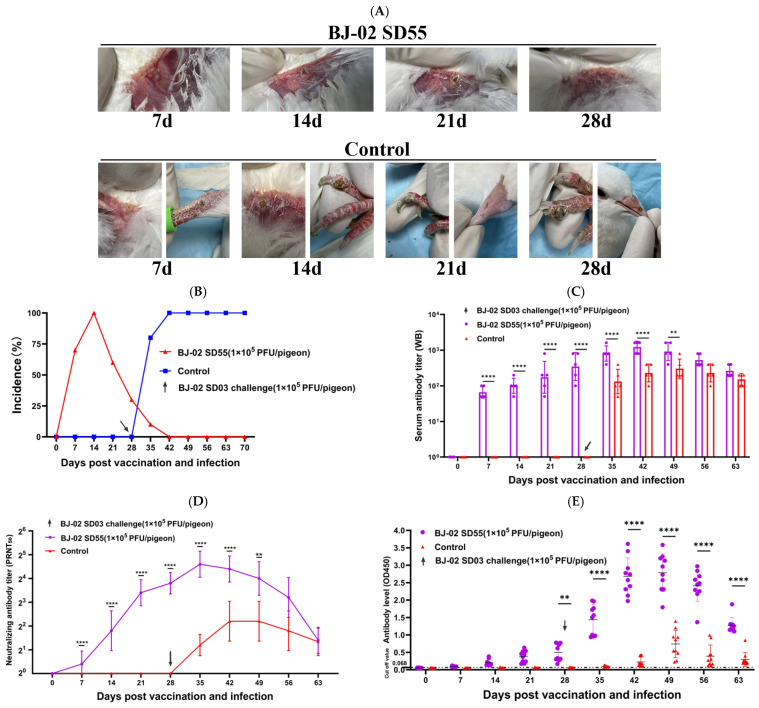
Dynamic monitoring of antibody after immunization and results of challenge protection test in squabs. (**A**) Pox on the body surface of pigeons in the immunized group and control group. (**B**) Incidence curve. (**C**) Detection of specific binding antibody titer against BJ-02 P4b protein by terminal dilution Western blot. The Y-axis uses a logarithmic scale to display the original titer. Statistical analysis was based on data transformed by log_10_. (**D**) Detection of serum neutralizing antibody titer by plaque reduction neutralization test (PRNT_50_). The Y-axis scale from 2^1^ to 2^6^ corresponds to neutralizing titers from 2 to 32, and 2^0^ represents no neutralizing activity detected. Statistical analysis was based on data transformed by log_2_. (**E**) Detection of specific antibody levels against BJ-02 P4b protein by indirect ELISA. The Y-axis represents the OD450nm absorbance value, and the dashed line indicates the Cut-off Value (0.068); pigeons were randomly divided into two groups (*n* = 10), and were inoculated with BJ-02 SD55 strain (10^5^ PFU/bird) or physiological saline (control group), respectively. On day 28 after inoculation with BJ-02 SD55 strain, a virulent challenge with BJ-02 SD03 strain (10^5^ PFU/bird) was performed (indicated by the black arrow in the figure). Data are presented as mean ± standard deviation. Statistical analysis was performed using two-way ANOVA and Sidak multiple comparison test (*p* < 0.01 for **, *p* < 0.0001 for ****).

Pigeons in the immunized group were inoculated by wing-web method with 1 × 10^5^ PFU/pigeon of the high-passage strain BJ-02 SD55, while the control group received 0.2 mL of normal saline. Specific binding antibodies were detected by Western blot and indirect ELISA at 7 days post-immunization, and antibody levels increased steadily thereafter. By the time of challenge (28 days post-immunization), the serum antibody titer reached a maximum of 1:1131 as detected by WB, and the maximum OD value measured by indirect ELISA was 0.7770. Neutralizing antibodies were first detected at 14 days post-immunization and rose steadily, with an average titer reaching 1:22.6 by day 28. The control group showed negative antibody results at all time points prior to challenge. At 28 days post-immunization, both groups were challenged with the virulent strain at the same dose. High levels of antibodies were detected as early as 7 days post-challenge, peaking between 7 and 14 days post-challenge, with the highest WB titer reaching 1:2263 and the highest ELISA OD value reaching 3.6143. Afterward, antibody levels gradually declined but remained high until day 63, with moderate antibody titers still detectable. Neutralizing antibodies peaked at 1:45.3 at 7 days post-challenge, then slowly decreased, remaining high until day 21 and tending toward negativity by day 35. The control group showed positive antibodies between 7 and 14 days post-challenge, but both binding and neutralizing antibody levels were significantly lower than those of the immunized group, with no notable upward trend in neutralizing antibodies, and they turned negative by day 63. Detailed results are shown in the figures (Figure 6C–E).

Simultaneously, cutaneous pock lesions on pigeons post-challenge were observed. In the immunized group, small specific pock lesions appeared at the inoculation site on day 7, which became larger, darker, and drier by day 14, clearly distinguishable from the surrounding normal skin. By day 21, the lesions dried further, with edges lifting and showing signs of shedding; no abnormalities were observed at other featherless sites. By day 28, the lesions had completely fallen off, and the skin at the affected area gradually recovered. In contrast, in the control group, pock lesions appeared at the inoculation site on day 7 post-challenge, with dried lesions visible on the claws. By day 14, the lesions at the inoculation site enlarged and showed signs of spreading and ulceration, and claw lesions increased in size and became ulcerated. By day 21, new pock lesions appeared on the beak, in addition to the previous sites. By day 28, the claw lesions had become larger, darker, and ulcerated, impairing walking, while the beak lesions also enlarged and darkened (Figure 6A). In summary, the high-passage BJ-02 SD55 provided 100% protective efficacy in the immunized pigeons. Following challenge with the virulent strain BJ-02 SD03, only pock lesions appeared at the inoculation site and disappeared within 4 weeks, with no lesions developing at other body sites, and the pigeons remained in good health. In contrast, control pigeons developed specific pock lesions sequentially at the inoculation site, claws, beak, and other featherless areas after challenge, which worsened over time without signs of recovery, accompanied by reduced water intake, decreased appetite, and deteriorating mental status.

## 4. Discussion

Our epidemiological survey results show varying degrees of PPV infection in Beijing (9.05%), Guangdong (16.11%), and Hainan (12.50%) between 2022 and 2023. A small-scale epidemiological study on avian poxvirus conducted by Lebdah et al. in 2019 [22] reported an overall positive rate of approximately 28%. Another survey, targeting different bird species, showed infection rates of 42.2% in chickens, 70.5% in turkeys, and 23.5% in canaries [23]. However, such epidemiological data remain limited, with inconsistent sampling types and geographical coverage. Thus, the overall positive rate observed in this study still requires further investigation. Nevertheless, a comparison of the positive rates across the three regions allows us to draw some preliminary conclusions: the positive rates in Guangdong and Hainan (16.11%) were slightly higher than in Beijing (9.05%), which likely reflects both geographical differences and the influence of environmental factors on PPV transmission. The hot and humid climate in Guangdong and Hainan supports the proliferation of hematophagous insects (e.g., Culex pipiens), which act as mechanical vectors and play a critical role in the rapid spread of the virus among pigeon populations [24]. Compared to other collected samples, the viral detection rate in scabs was particularly high (>60%), which is consistent with diagnostic findings for most avian poxvirus infections [25,26,27]. This result emphasizes the importance of appropriate sample selection for accurate diagnosis. The exceptionally high detection rate in scab samples confirms the strong epitheliotropism of poxviruses, which primarily replicate within keratinocytes and induce the formation of Bollinger bodies. This characteristic determines their primary mode of horizontal transmission, which occurs via mechanical injury or hematophagous insects [3,28,29]. Although no significant difference in breed susceptibility was observed between breeding and homing pigeons, the higher infection rate in squabs and juvenile pigeons aligns with the concept that pigeons aged 3–10 weeks are particularly vulnerable to viral infections due to the natural decline in maternal antibodies before the establishment of active immune responses [30]. As the number of passages increased, chorioallantoic membrane (CAM) lesions progressively worsened, evolving from focal pox lesions to diffuse thickening, edema, and congestion. By the third passage, marked edema and pale red changes indicated that the virus had achieved a high replication titer. This progressive enhancement of lesions demonstrates that the virus rapidly adapted to CAM tissue during serial passages, confirming its successful adaptation and efficient amplification within this host system [3].

Pigeon-derived isolates clustered in the same branch as *pigeonpox virus* reference strains from India and South Africa, indicating a very close genetic relationship with nucleotide homology exceeding 95%. This high degree of genetic similarity across continents suggested a potential genetic evolutionary connection between strains located far apart. Although direct epidemiological data is currently lacking, considering the range of pigeons and migratory birds, the possibility of cross-regional transmission of the pathogen through bird migration or international poultry trade cannot be ruled out [31,32]. Additionally, these isolates showed only 89.9–90.5% homology with fowlpox virus reference strains, reflecting a significant genetic distance, which may explain the poor efficacy of the fowlpox vaccine (subtype A1) in pigeon flocks under clinical conditions. Furthermore, the pigeon-derived isolates exhibited relatively distant relationships with passerine-origin canarypox virus, sparrowpox virus, and other clade B viruses, further confirming the distinct host specificity of avipoxviruses [10].

The complete genome sequence of the *pigeonpox virus* BJ-02 isolate was successfully obtained, with a contiguous sequence length of 280,269 bp. The BJ-02 genome consists of a central coding region flanked by inverted terminal repeats (ITRs) of 4691 bp in length. This genomic architecture, with a central coding region surrounded by ITRs, is consistent with structural features of other Avipoxviruses [26,33,34,35], and similarly exhibits an A + T-rich characteristic (70%). Comparative analysis with other Avipoxvirus sequences revealed that BJ-02 exhibits extremely high homology (>95%) with *pigeonpox virus* genomes isolated from South Africa (FeP2, NC_024447.1) and India (NC_036582.1). In contrast, the lowest similarity was observed with turkeypox virus (70.29%), and similarity with other avipoxvirus species ranged between 72.56% and 89.27%. According to poxvirus classification criteria, nucleotide sequence identity among strains within the same species typically exceeds 99%, while interspecies identity is approximately 96%. The high degree of conservation between BJ-02 and other *pigeonpox virus* isolates, along with significant sequence differences from other avipoxviruses (such as fowlpox virus), supports the classification of *pigeonpox virus* as a distinct species within the Avipoxvirus genus [36].

A total of 270 open reading frames (ORFs) were identified in the BJ-02 genome, exhibiting a high coding density of 90%. Similar to other sequenced avipoxviruses, BJ-02 retains 179 highly conserved genes [8,37]. However, among the predicted 270 ORFs, only 226 were annotated as complete genes, with the remainder including 15 truncated genes and 29 homologous genes in fragmented forms. This phenomenon of gene degradation, observed in South African avipoxviruses, is likely due to progressive mutations, genomic rearrangements, or sequence deletions, which reflect the adaptive evolution the virus undergoes during long-term evolution and under host selection pressure [7]. When compared to the closely related South African *pigeonpox virus* strain FeP2, BJ-02 shows notable differences in the composition of multigene families. The most significant difference lies in the ankyrin repeat gene family, where BJ-02 retains only 21 complete ankyrin repeat genes, while FeP2 has 26, with the missing genes largely present in truncated forms. Additionally, BJ-02 possesses only 3 complete V-type Ig domain family genes, while FeP2 has 4, with the missing gene present in a fragmented form. Previous studies suggest that the gain and loss of gene family members, such as ankyrin repeats in poxviruses, play a key role in host adaptation during long-term evolution. The abundance and integrity of these genes are not only associated with viral host tropism and immune evasion but also with the attenuation of virulence. Reductions in gene copy number or structural loss are often considered factors contributing to virulence attenuation. Therefore, the reduced number of complete ankyrin repeat genes and V-type Ig domain genes in the BJ-02 genome may suggest a relatively lower pathogenicity of this isolate, reflecting its unique host adaptation strategy under specific ecological conditions [10,38].

Furthermore, while previous studies have often detected natural integration of REV proviral sequences in the genomes of field-isolated fowlpox viruses [27,39,40], no REV-like sequences were found in the BJ-02 genome, although being limited by the inability to perform REV-specific PCR detection on the original clinical samples during the early stage of isolation. It is hard to prevent from excluding the possibility of sequence loss during in vitro passage. The current sequencing results objectively showed the genetic background of the isolate at the genomic level. This study preliminarily evaluated a high-passage pigeon pox virus strain, BJ-02 SD55, which is a potential vaccine candidate. Through continuous passage on CAM, PEF, and CEF, the high-passage strain BJ-02 SD55 showed signs of reduced virulence, manifested as milder CAM lesions (ranging from confluent lesions to scattered pox plaques), a slightly reduced plaque diameter, and differences in the distribution and severity of cutaneous pox lesions in inoculated pigeons. Meanwhile, after five consecutive passages in pigeons, no reversion to virulence was observed in the high-passage strain BJ-02 SD55, which preliminarily meets the safety requirements for live vaccines.

This strategy of obtaining highly attenuated strains through serial passage in vitro cell culture or chicken embryos is in line with the classical development pathway of modern safe poxvirus vaccines, such as the widely used modified vaccinia Ankara (MVA) strain [41,42]. Interestingly, the number of in vitro passages required for completed attenuation of avipoxviruses has varied considerably in the previous literature. Some studies suggest that to sufficiently attenuate highly pathogenic wild-type strains, serial passage in vitro may require 100 to 200 or even more passages; for example, the highly attenuated fowlpox virus FP9 strain, widely used as a recombinant vaccine vector, underwent over 400 serial passages before losing pathogenicity completely [38]. However, other studies have reported that attenuated strains of avipox virus with both high safety and good immunogenicity were selected after 40 passages of CEF [43]. In this study, the BJ-02 isolate showed attenuation potential after 50 passages, which may be attributed to the selection pressure of the in vitro culture system or the unique genetic background of BJ-02 (gene fragmentation), which could have allowed it to undergo adaptive evolution more readily and rapidly in vitro. However, this study still has limitations: firstly, there is a lack of genome sequencing alignment to pinpoint the specific mutations or deletions that led to attenuation; secondly, there is a lack of systematic natural transmission experiments to fully evaluate its biosafety. In the absence of molecular level evidence and horizontal transmission verification, the existing data alone is insufficient to conclude that it has been fully attenuated. Subsequent research needs to further confirm through genome sequencing alignment and natural transmission experiments that the strain has reached a stable attenuated state and meets biosafety standards.

## 5. Conclusions

This study analyzed the current prevalence of PGPV in several regions of China and conducted a preliminary evaluation of a candidate viral strain with potential for attenuation. The findings update the understanding of the current PGPV epidemiological status and provide strategic insights and candidate materials for addressing the challenges associated with cross-species vaccination in pigeons.

## Figures and Tables

**Table 1:** Antibody dynamic monitoring and challenge protection test protocol for squabs post-immunization.

Group	Inoculum	Dose	Immunization Route	Number	Immunization (Days)	Challenge (Days)	Challenge Dose
1	BJ-02 SD55	10^5^ PFU/pigeon	Wing-web inoculation	10	30	58	10^5^ PFU/pigeon
2	Physiological Saline	0.2 mL	Wing-web inoculation	10	30	58	10^5^ PFU/pigeon

## Data Availability

The data presented in this study are openly available. The complete genome sequence of a *pigeonpox virus* strain involved in this article has been uploaded to GenBank with accession number PX219506.

## Supplementary Materials

The following supporting information can be downloaded at: https://www.mdpi.com/article/10.3390/vetsci13040393/s1, Table S1: ORFs of *pigeonpox virus* BJ-02; Table S2: Number of ORFs in each of the 12 multigene families identified in the fully sequenced avian poxvirus genomes; Table S3: Details of Avipoxvirus used in this study.
