# Supplementary material for: First Complete Genome Sequencing of a Pigeonpox Virus Strain from Mainland China and Preliminary Evaluation of Its Attenuated Potential
Source: Vet Sci. 2026 Apr 17;13(4):393. doi: 10.3390/vetsci13040393 (PMC13119586; doi:10.3390/vetsci13040393)
Supplement: Supplementary file 1 [file vetsci-13-00393-s001.zip › vetsci-4214971-supplementary.pdf]

Table S1. ORFs of pigeonpox virus BJ-02

ORF	Start	End	strand	length (nt)	length (aa)	Predicated structure and/or function
PGPV001	496	1098	-	603	200	C-type lectin family protein
PGPV002	2118	2318	-	201	66	hypothetical protein
PGPV003	3527	4195	+	669	222	hypothetical protein
PGPV004	4497	4637	+	141	46	hypothetical protein
PGPV005	4848	4964	+	117	38	hypothetical protein
PGPV006	5945	6418	-	474	157	ankyrin repeat protein fragmented
PGPV007	6792	8024	+	1233	411	C4/C10L-like family protein
PGPV008	8880	10394	-	1515	504	ankyrin repeat protein
PGPV009	10425	10559	-	135	44	fragmented
PGPV010	11132	13174	-	2043	680	ankyrin repeat protein
PGPV011	13281	14348	-	1068	355	serpin family protein
PGPV012	14454	15335	-	882	293	alphaSNAP
PGPV013	15411	16967	-	1557	518	hypothetical protein
PGPV014	17102	17629	-	528	176	interleukin 10
PGPV015	17698	18690	-	993	331	ankyrin repeat protein
PGPV016	18791	20002	-	1212	403	ankyrin repeat protein
PGPV017	20087	21400	-	1314	437	ankyrin repeat protein
PGPV018	21814	22326	-	513	170	ankyrin repeat protein
PGPV019	22587	23300	-	714	237	hypothetical protein
PGPV020	23413	24150	-	738	245	V-type Ig domain protein
PGPV021	24213	26264	-	2052	683	ankyrin repeat protein
PGPV022	26435	26719	-	285	94	hypothetical protein
PGPV023	26747	27316	+	570	189	hypothetical protein
PGPV024	27313	28596	-	1284	427	C4/C10L-like family protein
PGPV025	28690	29685	+	996	331	G-protein-coupled receptor family protein
PGPV026	29700	31445	-	1746	581	ankyrin repeat protein
PGPV027	31521	32825	-	1305	434	ankyrin repeat protein
PGPV028	32891	34675	-	1785	594	ankyrin repeat protein
PGPV029	34807	35418	-	612	203	hypothetical protein
PGPV030	35473	36969	-	1497	498	ankyrin repeat protein
PGPV031	37196	37468	-	273	90	ankyrin repeat protein fragmented
PGPV032	37658	37792	+	135	44	hypothetical protein truncated
PGPV033	38164	39558	-	1395	464	ankyrin repeat protein
PGPV034	39622	40002	-	381	126	hypothetical protein
PGPV035	40108	42534	-	2427	808	alkaline phosphodiesterase
PGPV036	42661	43686	-	1026	341	alkaline phosphodiesterase
PGPV037	45123	45257	-	135	44	ankyrin repeat protein fragmented
PGPV038	45535	45942	-	408	135	hypothetical protein
PGPV039	46254	46748	-	495	164	hypothetical protein
PGPV040	46745	47182	-	438	145	dUTPase
PGPV041	47234	47761	-	528	175	Bcl-2
PGPV042	47809	48822	-	1014	337	serpin family protein
PGPV043	48855	49535	-	681	226	hypothetical protein
PGPV044	49622	51316	-	1695	564	DNA ligase
PGPV045	51351	52427	-	1077	358	serpin family protein

PGPV046	52469	53581	-	1113	370	nucleotide-sugar epimeras
PGPV047	53712	55442	-	1731	576	semaphorin
PGPV048	55892	56677	+	786	261	GNS1/SUR4
PGPV049	56754	57218	+	465	154	late transcription factor VLTF2
PGPV050	57239	58897	+	1659	552	rifampicin resistance N3L protein
PGPV051	58929	59798	+	870	289	mRNA capping enzyme
PGPV052	59876	61789	+	1914	637	NPH-1 transcription termination factor
PGPV053	61793	62470	-	678	225	muT motif expression regulator
PGPV054	62436	63149	-	714	237	muT motif
PGPV055	63419	64243	-	825	274	V-type Ig domain protein
PGPV056	64389	64874	-	486	161	RNA polymerase subunit RPO18
PGPV057	64861	66762	-	1902	633	early transcription factor VETF
PGPV058	66743	69118	-	2376	791	NTPase
PGPV059	70846	71466	-	621	206	CC chemokine family protein
PGPV060	71990	72319	-	330	109	CC chemokine family protein
PGPV061	72400	72999	-	600	199	CC chemokine family protein
PGPV062	73045	73701	-	657	218	uracil DNA glycosylase
PGPV063	73997	74344	-	348	115	hypothetical protein truncated
PGPV064	74320	74911	-	592	197	hypothetical protein truncated
PGPV065	75241	75645	+	405	134	glutathione peroxidase fragmented
PGPV066	75646	75978	+	333	110	hypothetical protein
PGPV067	75953	76369	-	417	138	hypothetical protein
PGPV068	76469	76750	-	282	93	HT motif family protein
PGPV069	77126	77536	-	411	136	hypothetical protein
PGPV070	77610	78416	-	807	268	virion protein
PGPV071	78512	79333	+	822	273	T10 protein
PGPV072	79348	79479	-	132	43	hypothetical protein
PGPV073	79731	80594	-	864	287	hypothetical protein
PGPV074	80613	80777	-	165	54	hypothetical protein fragmented
PGPV075	80850	81098	-	249	83	beta nerve growthfactor fragmented
PGPV076	81521	81841	-	297	98	interleukin 18-binding protein truncated
PGPV077	82320	82625	-	306	101	hypothetical protein
PGPV078	82629	83192	-	564	187	N1R/p28 family protein
PGPV079	83751	84128	+	378	125	Glutaredoxin
PGPV080	84101	84778	-	678	225	putative elongation factor
PGPV081	84772	85083	+	312	104	hypothetical protein
PGPV082	85110	86117	-	1008	335	transforming growth factor B
PGPV083	86189	88072	+	1884	627	metalloprotease
PGPV084	88056	90104	-	2049	682	DNA/RNA helicase/NPH-11
PGPV085	90137	91402	+	1266	421	virion core proteinase
PGPV086	91405	92580	+	1176	391	DNA-binding protein
PGPV087	92581	92826	+	246	81	IMV membrane protein
PGPV088	92836	93387	+	552	183	thymidine kinase
PGPV089	93455	93730	+	276	91	HT motif family protein
PGPV090	93761	94636	+	876	291	DNA-binding phosphoprotein
PGPV091	94637	94834	+	198	65	hypothetical protein
PGPV092	94841	95776	+	936	311	virion protein
PGPV093	95777	95881	+	105	35	hypothetical protein
PGPV094	95955	97925	+	1971	656	hypothetical protein

PGPV095	97867	98262	+	396	131	hypothetical protein
PGPV096	98259	98543	-	285	94	sulfhydryl oxidase ERV1
PGPV097	98570	101536	+	2967	988	DNA polymerase
PGPV098	101528	102376	-	849	282	hypothetical protein
PGPV099	102369	104084	-	1716	571	hypothetical protein
PGPV100	104210	109876	-	5667	1888	B22R family protein
PGPV101	109939	115426	-	5488	1829	B22R family protein
PGPV102	115669	121482	-	5814	1938	B22R family protein
PGPV103	121551	122099	+	549	182	RNA polymerase subunit RPO30
PGPV104	122152	124305	+	2154	717	hypothetical protein
PGPV105	124292	125710	+	1419	472	polyA polymerase large subunit PAP-L
PGPV106	125704	126048	-	345	114	DNA-binding virion core phosphoprotein
PGPV107	126125	126757	+	633	210	hypothetical protein
PGPV108	126876	127325	+	450	149	hypothetical protein
PGPV109	127534	127833	+	300	99	hypothetical protein
PGPV110	127889	133231	-	5343	1781	B22R family protein
PGPV111	133416	134549	+	1134	377	virion envelope protein
PGPV112	134585	136504	+	1920	639	virion release protein
PGPV113	136549	137785	+	1237	412	hypothetical protein
PGPV114	137960	139294	+	1335	444	SER/THR protein kinase
PGPV115	139269	139910	+	642	213	hypothetical protein
PGPV116	140002	140202	+	201	66	hypothetical protein
PGPV117	140684	141235	+	552	183	HAL3 domain protein
PGPV118	141315	141746	+	432	143	ankyrin repeat protein fragmented
PGPV119	141871	142053	+	183	61	ankyrin repeat protein fragmented
PGPV120	142597	142965	+	369	122	CC chemokine family protein
PGPV121	143452	144774	+	1323	440	hypothetical protein
PGPV122	144776	144967	+	192	63	RNA polymerase subunit RPO7
PGPV123	144967	145533	+	567	188	hypothetical protein
PGPV124	145498	146529	-	1032	343	virion core protein
PGPV125	147193	147804	+	612	203	hypothetical protein
PGPV126	148140	148859	+	720	239	hypothetical protein
PGPV127	150200	150835	+	636	211	thymidylate kinase
PGPV128	150890	151672	+	783	260	myristylated protein
PGPV129	151685	152695	+	1011	336	myristylated protein
PGPV130	152696	153427	+	732	243	myristylated protein
PGPV131	153462	153752	+	291	96	myristylated protein
PGPV132	153742	154647	-	906	301	hypothetical protein
PGPV133	154673	155434	+	762	253	DNA-binding virion core VP8
PGPV134	155435	155824	+	390	129	hypothetical protein
PGPV135	155775	156221	+	447	148	hypothetical protein
PGPV136	156254	157180	+	927	308	polyA polymerase PAPs
PGPV137	157177	157737	+	561	186	RNA polymerase subunit RPO22
PGPV138	157727	158140	-	414	137	membrane protein
PGPV139	158181	162044	+	3864	1287	RNA polymerase subunit RPO147
PGPV140	162050	162550	-	501	166	protein tyrosine phosphatase
PGPV141	162566	163138	+	573	190	hypothetical protein
PGPV142	163319	164320	-	1002	333	virion envelope protein (p35)
PGPV143	164321	166720	-	2400	799	RNA polymerase-associated protein RAP94

PGPV144	166870	167394	+	525	174	VLTF-4
PGPV145	167395	168345	+	951	316	DNA topoisomerase
PGPV146	168350	168808	+	459	152	putative 17kDa protein
PGPV147	168771	169031	-	261	86	hypothetical protein
PGPV148	169090	171648	+	2559	852	mRNA capping enzyme, large subunit
PGPV149	171757	171948	+	192	63	HT motif family fragmented
PGPV150	171997	172419	-	423	140	virion protein
PGPV151	172488	172625	+	138	46	fragmented
PGPV152	172772	173344	+	573	190	hypothetical protein
PGPV153	173412	174266	+	855	284	N1R/p28 family protein
PGPV154	174308	175024	+	717	238	Dck
PGPV155	175506	176135	+	630	209	hypothetical protein
PGPV156	176875	178107	+	1233	410	N1R/p28 family protein
PGPV157	178351	178749	+	399	132	HT motif family protein
PGPV158	178795	179781	+	987	328	N1R/p28 family protein
PGPV159	179836	181230	+	1395	464	Photolyase
PGPV160	181368	182108	+	741	246	N1R/p28 family protein
PGPV161	182189	182659	+	471	156	hypothetical protein
PGPV162	182707	183156	+	450	149	N1R/p28 family protein
PGPV163	183191	183592	+	402	133	N1R/p28 family protein
PGPV164	183642	183779	+	138	45	hypothetical protein
PGPV165	183795	184277	+	483	160	N1R/p28 family protein
PGPV166	184660	186435	+	1776	592	N1R/p28 family protein
PGPV167	186557	187327	+	771	256	N1R/p28 family protein
PGPV168	187967	188107	+	141	46	ankyrin repeat protein fragmented
PGPV169	188182	189333	+	1152	383	hypothetical protein
PGPV170	190664	191341	-	678	225	late transcription factor VLTF-3
PGPV171	191338	191556	-	219	72	virus redox protein
PGPV172	191572	193548	-	1977	658	virion core protein P4b
PGPV173	193632	194366	-	735	244	immunodominant virion protein
PGPV174	194405	194914	+	510	169	RNA polymerase subunit RPO19
PGPV175	194909	196033	-	1125	374	hypothetical protein
PGPV176	196040	198169	-	2130	709	early transcription factor large subunit VETF-L
PGPV177	198235	199140	+	906	301	intermediate transcription factor VITF-3
PGPV178	199102	199332	-	231	76	hypothetical protein
PGPV179	199333	202008	-	2676	891	virion core protein P4a
PGPV180	202026	202844	+	819	272	hypothetical protein
PGPV181	202845	203372	-	528	175	virion protein
PGPV182	203387	203536	-	150	50	hypothetical protein truncated
PGPV183	203558	203773	-	216	72	virion protein
PGPV184	203840	204115	-	276	92	virion envelope protein
PGPV185	204132	204239	-	108	36	virion envelope protein
PGPV186	204309	204602	-	294	98	hypothetical protein
PGPV187	204586	205695	-	1110	369	putative mystirilated membrane protein
PGPV188	205711	206307	-	597	198	phosphorylated virion membrane protein
PGPV189	206325	207713	+	1389	462	DNA helicase
PGPV190	207681	207947	-	267	88	hypothetical protein
PGPV191	207955	208296	+	342	114	hypothetical protein
PGPV192	208295	209593	+	1299	432	processivity factor

PGPV193	209593	210063	+	471	156	hypothetical protein
PGPV194	210073	211224	+	1152	383	intermediate transcription factor VITF-3
PGPV195	211251	214724	+	3474	1157	RNA polymerase subunit RPO132
PGPV196	214713	216539	-	1827	608	A-type inclusion protein
PGPV197	216575	217993	-	1419	472	A-type inclusion protein
PGPV198	217994	218416	-	423	140	hypothetical protein
PGPV199	218431	219339	-	909	302	RNA polymerase subunit RPO35
PGPV200	219314	219538	-	225	74	hypothetical protein
PGPV201	219594	219706	-	113	38	A30.5L-like protein
PGPV202	219716	220057	+	342	113	hypothetical protein
PGPV203	220058	220420	+	363	120	hypothetical protein
PGPV204	220409	221323	-	915	304	virion assembly protein
PGPV205	221498	222019	+	522	173	C-type lectin-like protein
PGPV206	222377	222697	+	321	106	V-type Ig domain fragmented
PGPV207	223134	223253	+	120	40	V-type Ig domain fragmented
PGPV208	223247	223423	+	177	59	V-type Ig domain fragmented
PGPV209	223462	224295	+	834	278	hypothetical protein
PGPV210	224350	225207	+	858	285	tyrosine protein kinase
PGPV211	225246	226274	+	1029	342	serpin family protein
PGPV212	226284	226946	-	663	220	hypothetical protein
PGPV213	227054	227980	+	927	308	G-protein-coupled receptor family protein
PGPV214	227991	228269	+	279	92	hypothetical protein
PGPV215	228350	228553	+	204	67	Beta-NGF protein truncated
PGPV216	229166	229747	+	582	194	hypothetical protein
PGPV217	229770	230159	-	390	129	HT motif family protein
PGPV218	231121	231492	+	372	123	epidermal growth factor-like protein
PGPV219	231495	232406	+	912	303	serine/threonine protein kinase
PGPV220	232437	232925	+	489	162	hypothetical protein
PGPV221	233000	233377	+	378	125	putative 13.7kDa protein
PGPV222	233469	233693	+	225	74	hypothetical protein
PGPV223	233867	234388	+	522	173	fragmented
PGPV224	234500	235384	+	885	294	ankyrin repeat protein
PGPV225	235435	235866	+	432	143	host range protein
PGPV226	236251	236379	+	129	43	hypothetical protein fragmented
PGPV227	236865	236978	+	114	37	ankyrin repeat protein truncated
PGPV228	237075	237647	+	573	190	ankyrin repeat protein truncated
PGPV229	237524	238078	+	555	185	ankyrin repeat protein truncated
PGPV230	238230	239552	+	1323	440	ankyrin repeat protein
PGPV231	239554	240105	-	552	183	A47L-like protein
PGPV232	240199	242442	+	2244	747	ankyrin repeat protein
PGPV233	242787	242921	+	135	45	ankyrin repeat protein fragmented
PGPV234	243391	243711	+	321	107	ankyrin repeat protein fragmented
PGPV235	244086	244967	+	882	293	serine/threonine protein kinase
PGPV236	245036	246121	+	1086	361	ankyrin repeat protein
PGPV237	246447	246698	+	252	83	ankyrin repeat protein fragmented
PGPV238	247067	247186	+	120	39	ankyrin repeat protein fragmented
PGPV239	247420	247725	+	306	101	ankyrin repeat protein fragmented
PGPV240	247757	248716	+	960	319	fragmented
PGPV241	248766	250277	+	1512	503	ankyrin repeat protein

PGPV242	251299	251856	-	558	185	putative A47L-like protein
PGPV243	251931	252086	+	156	51	ankyrin repeat protein fragmented
PGPV244	252343	252765	+	423	140	ankyrin repeat protein fragmented
PGPV245	253067	253465	+	399	133	ankyrin repeat protein fragmented
PGPV246	253539	254984	+	1446	481	ankyrin repeat protein
PGPV247	255028	256536	+	1509	502	ankyrin repeat protein
PGPV248	257205	257447	+	243	81	ankyrin repeat protein fragmented
PGPV249	257529	257703	+	175	58	ankyrin repeat protein fragmented
PGPV250	257729	257854	+	126	41	ankyrin repeat protein fragmented
PGPV251	258241	259080	+	840	279	N1R/p28 family protein
PGPV252	258928	259296	-	369	122	hypothetical protein
PGPV253	259602	259821	+	220	73	C-type lectin-like protein fragmented
PGPV254	259965	261200	+	1236	412	ankyrin repeat protein
PGPV255	261752	262381	+	630	210	Ig domain protein
PGPV256	262433	264319	+	1887	628	ankyrin repeat protein
PGPV257	264354	264932	+	579	192	ankyrin repeat protein
PGPV258	265834	265998	+	165	54	Ig domain protein fragmented
PGPV259	266319	268322	+	2004	668	hypothetical protein
PGPV260	268375	268602	-	228	75	ankyrin repeat protein fragmented
PGPV261	269348	270031	+	684	227	Ig domain protein truncated
PGPV262	270069	270776	+	708	235	Ig domain protein truncated
PGPV263	270872	272629	+	1758	585	ankyrin repeat protein
PGPV264	272687	273055	+	369	122	Efc family protein
PGPV265	273173	273622	+	450	149	N1R/p28 family protein
PGPV266	274843	275166	+	324	107	hypothetical protein
PGPV267	275235	275609	+	375	124	C-type lectin family protein
PGPV268	276074	276742	-	669	222	hypothetical protein
PGPV269	277951	278151	+	201	66	fragmented
PGPV270	279171	279773	+	603	114	C-type lectin-like protein

Table S2.Number of ORFs in each of the 12 multigene families identified in the fully sequenced avian poxvirus genomes.

Gene family	BJ-02	No.20 TW2022	PPV/Pur	FeP2	PEPV	PEPV2	FWPV	TKPV	MPPV	MPPV2	CNPV	SWPV1	SWPV2	MLPV	ALPV	FGPV
Ankyrin Repeat	23	24	26	26	33	49	31	16	62	78	51	50	46	47	48	45
B22R	4	4	4	4	5	6	6	1	7	9	6	6	7	7	6	4
N1R/p28	11	11	11	11	11	24	10	3	24	20	26	20	20	25	28	13
C4L/C10L	2	2	2	2	2	3	3	2	2	4	3	2	3	3	3	2
CC chemokine	4	4	4	4	1	5	4	2	4	7	5	6	5	5	5	6
C-type lectin	4	4	4	4	7	11	9	2	10	11	11	13	11	13	14	4
G-protein-copuled receptor	2	2	2	2	3	4	3	2	4	4	4	4	4	4	4	3
HT motif	4	4	4	4	5	5	6	1	5	5	5	4	4	5	5	7
Ig-like domain	3	4	3	4	6	9	5	3	10	13	9	9	8	8	9	9
Serpin	4	4	4	4	4	5	5	3	5	5	5	5	5	5	5	5
Efc	1	1	1	1	1	2	3	1	2	3	2	2	2	1	2	1
TGF-β	1	1	1	1	1	5	1	1	4	5	5	3	4	6	5	1

Table S3.Details of Avipoxvirus used in this study.

Virus	Submission date	Origin	Length/bp	Accession number
Fowlpox virus	2023	USA	288539	NC_002188
Fowlpox virus	2018	USA	295938	MH734528
Fowlpox virus	2018	USA	291057	MH709125
Fowlpox virus	2018	USA	288196	MH719203
Fowlpox virus	2018	USA	298798	MH709124
Fowlpox virus	2000	USA	288539	AF198100
Fowlpox virus	2018	China	308827	MG702259
Fowlpox virus	2020	China	282402	KX196452
Fowlpox virus	2022	Republic of korea	295688	MW558076
Fowlpox virus	2022	Republic of korea	297405	MW558073
Fowlpox virus	2022	Republic of korea	297159	MW558068
Fowlpox virus	2022	Republic of korea	288501	MW558081
Fowlpox virus	2022	Republic of korea	278618	MW558079
Fowlpox virus	2022	Republic of korea	287251	MW558078
Fowlpox virus	2022	Republic of korea	291194	MW558077
Fowlpox virus	2022	Republic of korea	292650	MW558080
Fowlpox virus	2022	Republic of korea	297250	MW558072
Fowlpox virus	2021	Australia	298250	OK558609
Fowlpox virus	2021	Australia	293578	MW142017
Fowlpox virus	2018	France	288539	MF766432
Fowlpox virus	2018	France	288539	MF766431
Fowlpox virus	2018	France	288539	MF766430
Canarypox virus	2023	USA	359853	NC005309
Canarypox virus	2003	USA	359853	AY318871
Shearwaterpox virus	2017	Australia	326929	KX857216
Shearwaterpox virus	2017	Australia	351108	KX857215
Pigeonpox virus	2025	China	280269	PX219506
Pigeonpox virus	2014	South Africa	282356	KJ801920
Pigeonpox virus	2023	Indian	280058	ON375849
Pigeonpox virus	2026	China	293900	PV250209
Penguinpox virus	2014	South Africa	306862	KJ859677
Penguinpox virus	2021	Australia	349821	MW296038
Flamingopox virus	2017	SouthAfrica	293123	MF678796
Magpiepox virus	2019	Australia	293226	MK903864
Magpiepox virus	2025	Australia	298392	MW485973
Mudlarkpox virus	2021	Australia	342723	MT978051
Turkeypox virus	2020	USA	188534	NC_028238
Crowpox virus	2022	Australia	328768	ON408417
